# Efficient Generation of *Myostatin (MSTN)* Biallelic Mutations in Cattle Using Zinc Finger Nucleases

**DOI:** 10.1371/journal.pone.0095225

**Published:** 2014-04-17

**Authors:** Junjie Luo, Zhiyuan Song, Shengli Yu, Dan Cui, Benli Wang, Fangrong Ding, Song Li, Yunping Dai, Ning Li

**Affiliations:** 1 State Key Laboratory for Agrobiotechnology, College of Biological Science, China Agricultural University, Beijing, People’s Republic of China; 2 Beijing GeneProtein Biotechnology Co., Ltd., Beijing, People’s Republic of China; 3 College of Animal Science, Yunnan Agricultural University, Kunming, People’s Republic of China; Imperial College London, United Kingdom

## Abstract

Genetically engineered zinc-finger nucleases (ZFNs) are useful for marker-free gene targeting using a one-step approach. We used ZFNs to efficiently disrupt bovine *myostatin* (*MSTN*), which was identified previously as the gene responsible for double muscling in cattle. The mutation efficiency of bovine somatic cells was approximately 20%, and the biallelic mutation efficiency was 8.3%. To evaluate the function of the mutated *MSTN* locus before somatic cell nuclear transfer, MSTN mRNA and protein expression was examined in four mutant cell colonies. We generated marker-gene-free cloned cattle, in which the *MSTN* biallelic mutations consisted of a 6-bp deletion in one of the alleles and a 117-bp deletion and 9-bp insertion in the other allele, resulting in at least four distinct mRNA splice variants. In the *MSTN* mutant cattle, the total amount of MSTN protein with the C-terminal domain was reduced by approximately 50%, and hypertrophied muscle fibers of the quadriceps and the double-muscled phenotype appeared at one month of age. Our proof-of-concept study is the first to produce *MSTN* mutations in cattle, and may allow the development of genetically modified strains of double-muscled cattle.

## Introduction

Myostatin (MSTN), also named growth/differentiation factor-8, is a member of the transforming growth factor-β superfamily, and is a negative regulator of muscle growth [1], [2]. Previous studies of mutations in *MSTN* and *MSTN* knockouts have shown that the inhibition of MSTN expression causes double muscling in cattle, a phenotype that is characterized by a substantial increase in skeletal muscle mass [1], [3]–[8]. The Belgian Blue and Piedmontese cattle breeds, which have a muscle mass approximately 20% greater than that of other cattle breeds, have natural mutations in *MSTN* [5], [6], [9]. Thus, selective breeding for specific *MSTN* mutations might result in increased muscle mass and greater commercial value.

Relying on natural mutations for selective animal breeding is most often impractical because they occur randomly and at low frequencies and require long-term phenotype screening. The use of conventional hybridization breeding for introducing pre-existing mutations is also time-consuming, especially in large, genetically complex domestic animals. The use of RNAi technology to reduce gene expression is unreliable because of position effect, the inheritability of transgenes, and gene instability. Classical gene-targeting technology provides another option for introducing mutations. However, the low efficiency and unavailability of embryonic stem cells limit its application in livestock. Therefore, a method to quickly and easily introduce specific mutation into domestic animal populations might be useful for improving beef cattle breeds.

The high efficiency of zinc-finger nuclease (ZFN)-mediated gene targeting has been demonstrated in multiple species, and has renewed optimism among researchers seeking to develop methods for fine-scale genetic modifications in domestic animals [10]–[16]. ZFNs induce double-strand DNA breaks at specific sites in the chromosomes of cells [17], which are repaired through host-cell mechanisms, including homologous recombination and non-homologous end-joining (NHEJ) repair processes, to maintain chromosome integrity. NHEJ is a major DNA repair pathway in eukaryotes that is error-prone and causes gene mutations consisting of short fragment deletions or insertions [18]. Therefore, the combination of ZFN-induced gene targeting and somatic cell cloning can facilitate the manipulation of gene function in domestic animals.

In our current study, we introduced specific *MSTN* mutations in Chinese yellow cattle to increase muscle mass. The open reading frame (ORF) of the *MSTN* gene of bovine fibroblasts was disrupted through NHEJ-mediated DNA repair of ZFN-induced cleavage. The levels of MSTN mRNA and protein in four different mutant colonies were examined to identify clones with reduced MSTN expression. We produced marker-gene-free *MSTN* biallelic mutant cattle using somatic cell nuclear transfer (SCNT). Our results show that the ZFN-based method induced *MSTN* disruptions with high efficiency, and the levels of MSTN mRNA and protein were significantly lower in the cattle with the double-muscled phenotype.

## Results

### *MSTN* Mutation in Bovine Primary Fibroblasts Induced by ZFNs

**Figure 1:**
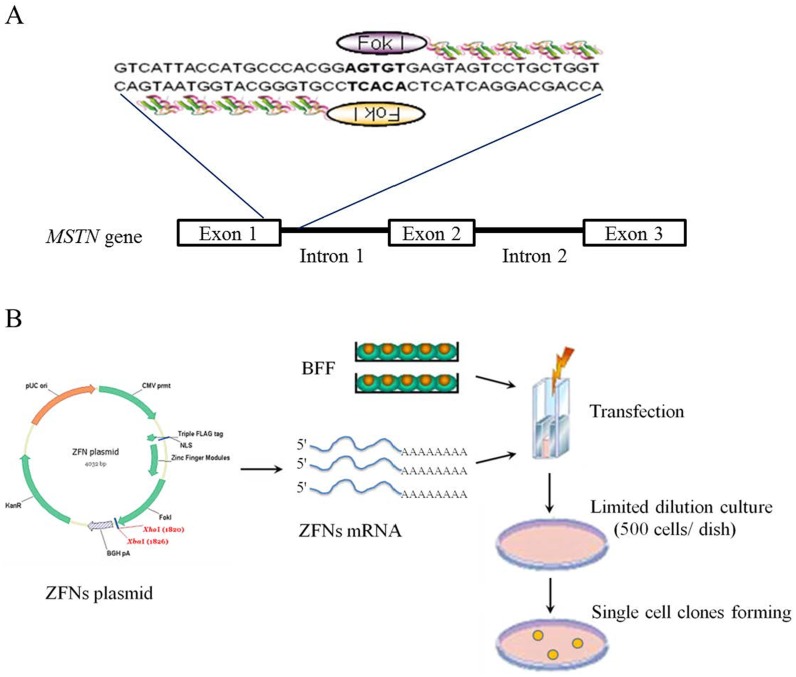
Use of ZFNs to disrupt bovine *MSTN*. (A) The target sequences of engineered ZFNs at the *MSTN* locus. The specific recognition sequences of the ZFNs included 19 bp upstream and 16 bp downstream of the *Fok*I non-specific cleavage sequence (AGTGT or ACACT). (B) Flowchart depicting the methodology used to generate ZFN-induce mutations in the single-cell colonies without drug selection. The mRNA was transcribed from the MSTN*-*ZFN plasmid in vitro, and the BFF cell line was derived from a Chinese domestic yellow cattle fetus. The BFF cells were transfected with MSTN-ZFN mRNA using the Neocleofector reagent for 24 to 48 h. Limiting dilution was used to form single-cell colonies at a cell concentration of approximately 500 cells/dish (10 cm^2^). The single-cell colonies were generated after culturing for an additional 6 to 7 days.

The *MSTN*-specific ZFNs (MSTN*-*ZFNs) were designed and assembled by Sigma-Aldrich (St. Louis, MO, USA). The pair of ZFNs with the highest gene-targeting efficiency in bovine primary fibroblasts were selected for our study (Table S1). One ZFN recognized a 19-bp sequence in exon 1 that flanked the 5-bp cleavage site, and the other ZFN recognized a 16-bp sequence in intron 1 that flanked the cleavage sequence downstream (Figure 1A). The cleavage site was located near the splicing signal sequence in the ORF, and the repaired nucleotide sequence was expected to cause the gene product to be dysfunctional. Single-cell colonies were generated by limiting dilution in drug-free cell culture medium, and the cells were propagated for further analysis (Figure 1B).

The PCR amplicons of mixed cells cultured for 24 h after transfection with MSTN-ZFNs mRNA were examined using the Surveyor nuclease assay to detect mutation/disruption of the ZFN targeted sites. As shown in Figure S1, the majority of the PCR product was approximately 760 bp in length, and two fragments approximately 560 bp and 200 bp were observed after digestion by the Surveyor nuclease, indicating that the pair of ZFNs cut the target DNA sequence with high efficiency (≥5%). The PCR amplicons were TA-cloned, and sequenced to further assess the mutation and targeting efficiency of the MSTN-ZFNs. We tested the same loci targeted by the MSTN-ZFNs in different bovine cell lines, and similar gene disruption efficiencies were observed (Table S2).

**Figure 2:**
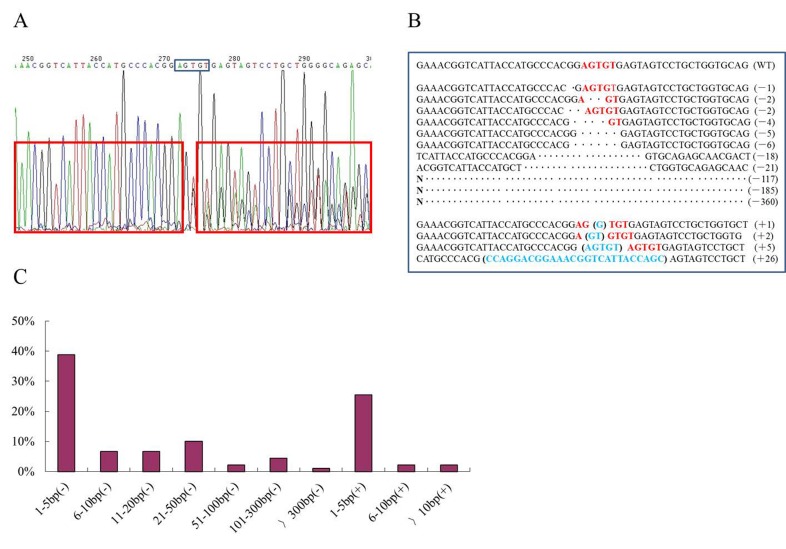
Analysis of *MSTN* disruption at the ZFN-targeted site. (A) Direct sequencing of the *MSTN* PCR amplicons from single-cell colonies. There were double peaks to the right of the ZFN (*Fok*I) target cleavage locus (AGTGT), indicating that *MSTN* mutation (deletion or/and insertion) had occurred. (B) Sequence alignment of ZFN-induced deletions (top) and insertions (bottom) in *MSTN* in bovine fibroblasts. Each sequence represented an individual allele. The red letters are ZFN cleavage sites, and the blue parenthetical letters represent insertions. (C) Distribution of different *MSTN* mutation types. Of the 90 mutations analyzed, the most of the mutations consisted of short-fragment (1–5 bp) deletions (–) or insertions (+), with more deletions occurring than insertions. The *y* axis represents the percentage of *MSTN* mutations and the *x* axis represents the type of mutation.

The PCR products from single-cell colonies were sequenced directly. If a mutation (deletion or/and insertion) in *MSTN* was induced by ZFN cleavage, there would be double peaks in the chromatogram to the right of the cleavage sequence (AGTGT) (Figure 2A). The PCR products of single-cell colonies with double peaks were TA-cloned, and sequenced to characterize the mutation. Following transfection with the MSTN-ZFN mRNAs, 60 single-cell colonies were examined to detect mutations. Mutations were identified in 12 (20%) of the colonies, five (8.3%) of which had biallelic mutations (Table 1). These mutation efficiencies were higher than those of transfections using MSTN-ZFN expression plasmids (17.8% and 4.4%, respectively). In cell colony 5, one mutation was identified, and no wild-type (WT) allele was observed, suggesting that the repaired gene sequences were the same on both homologous chromosomes. However, in most of the mutant cell colonies, the repaired allele sequences were different. Most of the mutations were short-fragment deletions or insertions (Figure 2B, 2C), which is consistent with a previous report of ZFN-induced mutagenesis [13]. Previous studies have shown that ZFN dimers cut spacers of different lengths, and produced 5′ overhangs of varying lengths [19]–[21]. Other studies showed that ZFNs spaced 6 bp apart produced 4-bp 5′ overhangs in most cases [12], whereas ZFNs spaced 5 bp apart produced 5-bp 5′ overhangs in 93% of the cleavage events examined [22]. The ZFNs used in our study were spaced 5 bp apart, and produced mutations consisting of 5-bp deletions or insertions, indicating that ZFNs spaced 5 bp apart produce 5-bp 5′ overhangs in most cases (Table S3).

**Table 1:** Comparison of gene mutations induced using MSTN-ZFN mRNA (set 1) and plasmid DNA in single-cell colonies.

Cell line	ZFNs construct	Mutation	Cell colonies	Mutation efficiency*	Biallelic mutation efficiency*
LXH-LS	mRNA	12	60	20.00%	5/60 (8.33%)
LXH-LS	plasmid	16	90	17.78%	4/90 (4.44%)

*the mutant efficiency was calculated by mutant colonies/total single cell colonies.

To examine the fidelity of the ZFNs, we compared the ZFN induced-mutation efficiency in different species with similar sequences at the target site. The bovine-fibroblast, sheep-fibroblast, pig-fibroblast, human-HT1080, rabbit-fibroblast, and mouse-C2C12 cell lines were transfected the *MSTN*-ZFN mRNAs (set 1). As shown in Table 2, the three mismatched nucleotides in the sheep *MSTN* target site caused a dramatic change in ZFNs cutting efficiency.

**Table 2:** The mutation efficiency of MSTN-ZFN (set 1) in different mammalian species.

Species	Target sequence	Mismatch (bp)	Mutation*
Bovine	GTCATTACCATGCCCACGG***AGTGT***GAGTAGTCCTGCTGGT	0	17/117 (14.53%)
Sheep	GTCATTACCATGCCCACGG***AGTGT***GAGTAGTtCTGCTaGg	3	1/96 (1.04%)
Pig	aTgATTACCATGCCtACaG***AGTGT***aAGTAGTCCTGtTaGT	7	0/108
Human	aTCATTACCATGCCtACaG***AGTGT***aAGTAGTCCTatTaGT	7	0/99
Rabbit	GgaAacACtATGCCtACcG***AGTGT***aAGTAtaCCTGtaaGT	13	0/103
Mouse	caCgcTACCATGCCtACaG***AGTGT***aAGTAtatCTGtTaaa	14	0/96

*The mutant efficiency is calculated by the mutant TA-cloning of PCR products of mixed cells/total sequencing number. The mismatch affected the ZFNs cut-efficiency dramatically. Lowercase letters were represented different nucleotides according to the targeting sequence of bovine. The bold italics represent the spacer nucleotides between the two ZFN monomers.

### MSTN mRNA and Protein Expression in Mutant Cell Colonies

**Figure 3:**
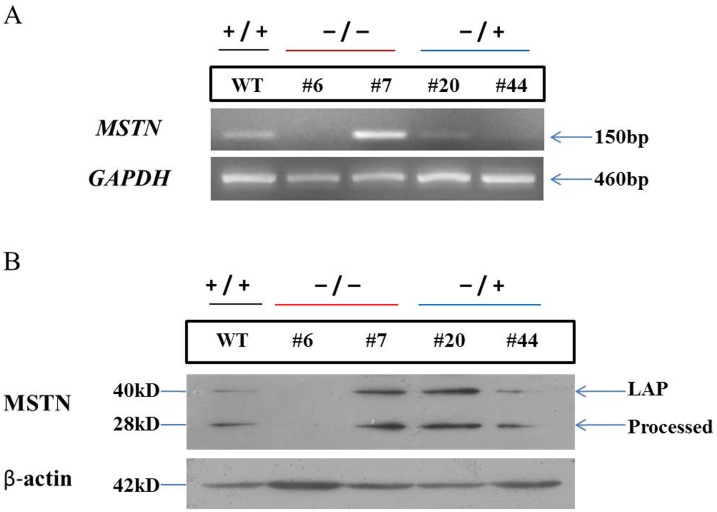
Analysis of *MSTN* mRNA and N-terminal protein in mutant cell colonies. (A) RT-PCR analysis of mRNA transcribed from *MSTN* mutations in bovine fibroblasts. Among the biallelic mutant colonies, the MSTN mRNA was not expressed in colony 6, but was highly expressed in colony 7. Among the monoallelic mutant colonies, the expression of MSTN mRNA in colonies 20 and 44 was reduced. The WT *MSTN* allele (+) and/or mutant *MSTN* allele (–) are indicated at the top. *GAPDH* was used as a loading control. (B) Western blot analysis of *MSTN* mutants in bovine fibroblasts. The N-terminal MSTN was not expressed in colony 6, but was highly expressed in colony 7 and 20, compared with that in the WT cells. Total protein (50 µg) from bovine fibroblasts was subjected to SDS-PAGE on a 12% acrylamide gel, and the N-terminal MSTN was detected using a mouse anti-myostatin antibody. Latency-associated peptide (LAP) and the processed form of N-terminal MSTN are indicated. β-actin was used as a loading control.

To evaluate the function of the mutated *MSTN* loci before SCNT, the expression of MSTN mRNA and protein in two colonies containing biallelic *MSTN* mutations, two colonies containing monoallelic *MSTN* mutations, and one WT colony was examined using RT-PCR (Table S4). Among the biallelic *MSTN* mutants, colony 6 had a 117-bp deletion and 9-bp insertion in one allele and a 6-bp deletion in the other allele, and colony 7 had 2- and 4-bp deletions. Among the monoallelic *MSTN* mutations, colonies 20 and 44 had 4- and 2-bp deletions, respectively. Although both had biallelic mutations, the expression of MSTN mRNA was not detected in the cells from colony 6 (Figure 3A), whereas the level of MSTN mRNA in the cells of colony 7 was higher than that of the WT cells. In the cells of colonies 20 and 44 (monoallelic mutations), the level of MSTN mRNA was significantly lower than that in the control cells.

The level of MSTN protein in the cells of colonies 6, 7, 20, and 44 was compared to that of the WT cells using western blotting. As shown in Figure 3B, latency-associated peptide (40 kDa) and the processed form of the N-terminal domain of the MSTN protein (28 kDa) was detected in the cells of all of the colonies, except colony 6, in which neither protein was detected, which is consistent with the RT-PCR data. However, although the level of MSTN mRNA in the cells of colonies 20 and 44 was lower than that of the WT cells, and the level of N-terminal domain of the MSTN protein in the cells of colony 20 was higher than that in the WT cells (Figure 3B). The level of the N-terminal domain of the MSTN protein was also higher in the cells of colony 7, compared with that of the WT cells, which was consistent with the RT-PCR data.

### *MSTN* Knockout in Cloned Calves

Cells with *MSTN* mutations were selected for SCNT, and cloned blastocysts were transferred to synchronized recipients. At 90 days post-transfer, 35 recipients were pregnant, and 18 of the fetuses were carried to term (Table S5). The physiological and biochemical indexes of two of the cloned calves were monitored, and no untoward effect of the *MSTN* mutations were observed.

**Figure 4:**
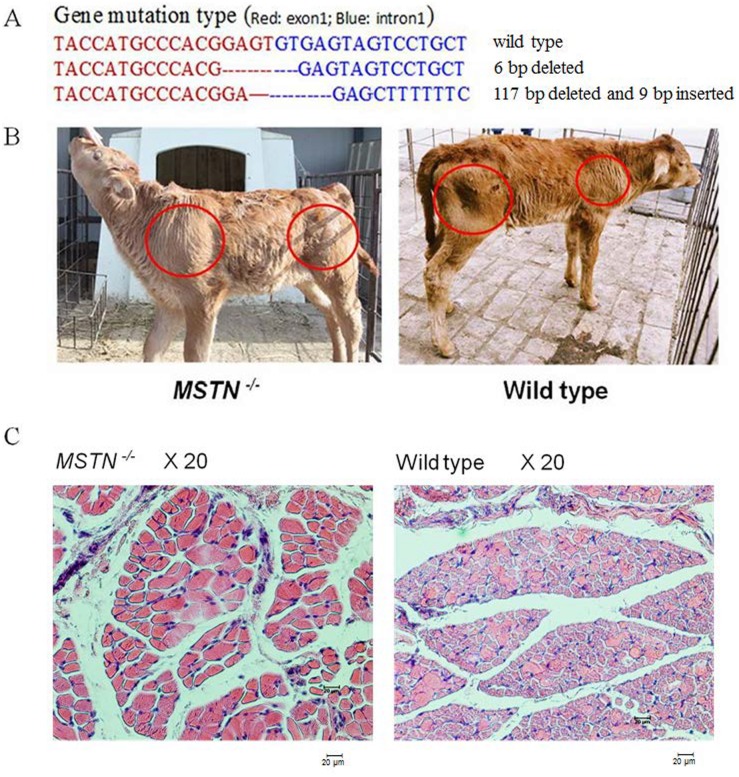
Characterization of *MSTN* gene knockout in cloned cattle. (A) Sequencing analysis of *MSTN* biallelic mutation types in cloned calves. The *MSTN* biallelic mutation types consisted of a 6-bp deletion in one allele (the last 4 bp of exon 1 and the first 2 bp of intron 1) and a 117-bp deletion (nucleotide positions 8–124 in intron 1) and a 9-bp insertion (the last 2 bp of exon 1 and the first 7 bp of intron 1, AG GCACGGG) in the other allele, which were consistent with colony 6. The red letters represent *MSTN* exon 1, and the blue letters represent *MSTN* intron 1. (B) Calves with *MSTN* biallelic mutations displayed the doubled-muscled phenotype, and exhibited no untoward effects. In red circles, the muscle mass in *MSTN* mutant (left) was greater than that of the wild-type calf (right). (C) Hematoxylin and eosin -stained cross-sections of the quadriceps muscle. Muscle fibers from calves with *MSTN* biallelic mutations (left) were hypertrophic, compared to those of the wild-type calf (right). All animals were one month old on the date of the tissue sample collection.

The DNA sequencing indicated that the *MSTN* biallelic mutations in both calves each consisted of a 117-bp deletion (nucleotide positions 8 to 124 in intron 1) and a 9-bp insertion (last 2 bp of exon 1 and first 7 bp of intron 1) in one allele and a 6-bp deletion (last 4 bp of exon 1 and first 2 bp of intron 1) in the other allele (Figure 4A). Both of the mutant calves exhibited the double-muscled phenotype at one month of age (Figure 4B), and histological examination showed muscle fiber hypertrophy, relative to that of the WT control calf (Figure 4C).

To assess the off-targeting effect of the ZFNs in the *MSTN* mutant cattle, similar target sequences were predicted using BLASTn. The genomic DNA of the mutant calves was analyzed by PCR and DNA sequencing using primer pairs that flanked 15 of the most similar target sequences (Table S6). None of the similar target sequences were mutated, relative to those of the control calf (Table S7), indicating that the ZFNs in set 1 exhibited a high level of specificity (a low rate of off-targeting) in the bovine cells.

### MSTN mRNA and Protein Expression in Cloned Calves

**Figure 5:**
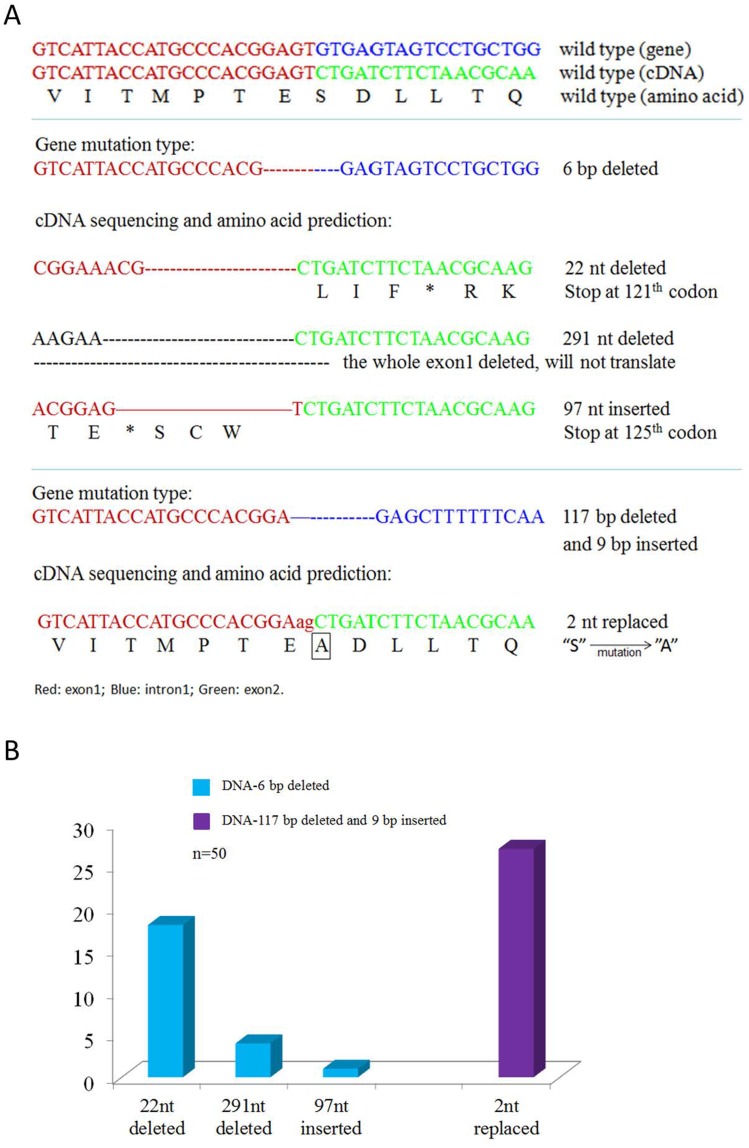
MSTN mRNA sequencing and protein prediction in cloned cattle. (A) The 6-bp deletion in the splicing signal sequence in the *MSTN* ORF, which comprised one allele, resulted in three mRNA splice variants, in which a 22 nt deletion, a 291 nt deletion, or 97 nt insertion occurred, all of which caused the premature termination of translation (22 nt deletion, 97 nt insertion) or prevented translation completely (291 nt deletion). The 117-bp deletion and 9-bp insertion that comprised the other allele produced only one type of transcript, in which 2 consecutive nucleotide substitutions occurred, which resulted in one amino acid substitution (serine to arginine) in the MSTN protein. The red, blue, and green letters represent the sequences of exon 1, intron 1, and exon 2 of *MSTN*, respectively. The asterisk indicates the stop codon. (B) The frequencies of the mRNA splice variants. The mRNA frequency of the dinucleotide substitution was highest, followed by the 22-nt deletion, the 291-nt deletion, and the 97-nt insertion. The overall frequencies of the two alleles were equivalent.

To examine the effect of the *MSTN* mutations on MSTN mRNA expression, complementary DNA (cDNA) was generated from the MSTN mRNA, and the cDNA was sequenced. The *MSTN* mutation caused by the 6-bp deletion disrupted the splicing signal sequence in the ORF, which resulted in the production of at least three mRNA splice variants, resulting in a 22- or 291-nt deletion or a 97-nt insertion in the MSTN mRNA. The frameshift mutation caused by the 22-nt deletion or the 97-nt insertion in the mRNA resulted in premature termination of translation, and the 291-nt deletion prevented translation completely (Figure 5A). Of the three types of frameshift mutations, the 22-nt mRNA deletion occurred most frequently, and the 97-nt mRNA insertion was rarest (Figure 5B). The *MSTN* mutation caused by the 117-bp deletion and 9-bp insertion resulted in two consecutive base substitutions in the MSTN mRNA that caused one amino acid substitution (Figure 5A). The frequencies of the two *MSTN* mutant alleles were equivalent (Figure 5B).

**Figure 6:**
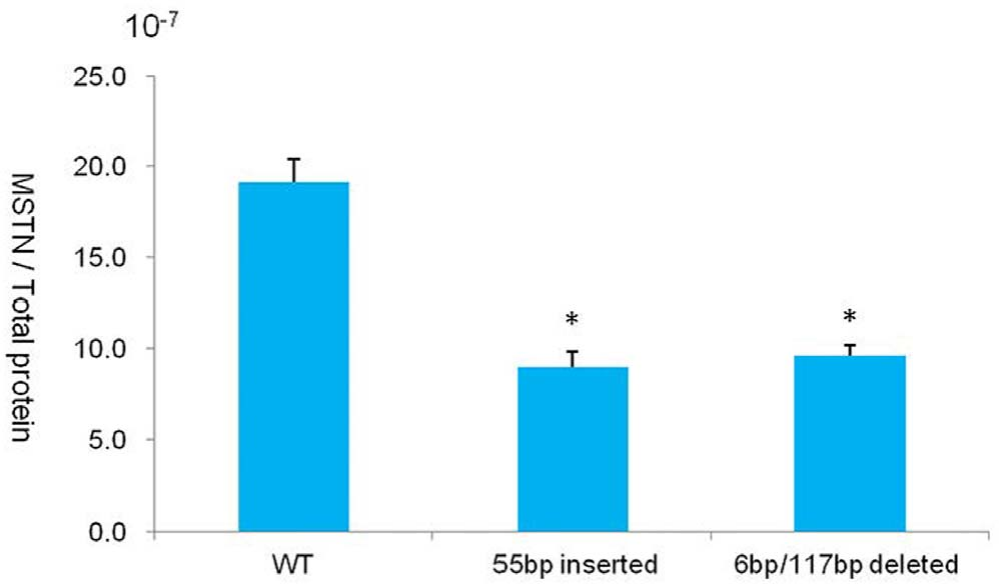
ELISA analysis of MSTN protein with a functional C-terminal domain in cloned cattle. We also produced a healthy cloned calf with monoallelic *MSTN* mutation, which consisted of a 55-bp insertion in intron 1, and caused the premature termination of translation. This allele is represented by “55 bp inserted” in the chart, and “6 bp/117 bp deleted” represents the double-muscled cloned bovine previously discussed in Figure 4. The level of MSTN protein with a functional C-terminal domain was reduced by approximately 50% in both mutant calves, compared to that of the WT calf. The ELISA data were analyzed using paired Student’s *t*-tests. The error bars represent the standard deviations of three experiments (*P<0.05 indicates a statistically significant difference compared to the WT calf).

We also assessed the level of MSTN mRNA and protein using RT-PCR (Figure S2), western blotting (Figure S3), and ELISA. We found that the level of MSTN mRNA in the cells of the mutant calves was less than that in the cells of the WT calves, and the level of the N-terminal domain of the MSTN protein was higher in the cells of the mutant calves, compared to that in the cells of the WT calves. The levels of the mRNA of the MSTN signaling pathway related factors, *P21, Myf5*, and *Myogenin*, were also examined. The RT-PCR results indicated that the level of the P21 and Myf5 mRNAs in the cells of the mutant calves were lower than those of the WT calves, whereas the level of the Myogenin mRNA was higher in the cells of the mutant calves, compared to that of the WT calves (Figure S2).The ELISA analysis indicated that the level of MSTN protein with a C-terminal domainin the cells of the mutant calves was approximately 50% lower than that of the WT calves (Figure 6), which is consistent with the results of the mRNA analysis (Figure 5A). These results suggest that the double-muscled phenotype of the cloned mutant calves was caused by a decrease in the level of MSTN protein with the C-terminal domain, which is consistent with previous reports that the mutation or knockout of *MSTN* increased muscle mass [1], [5], [6], [9], [23]. We will perform a more detailed phenotypic analysis of numerous offspring of these cloned cattle in the future.

## Discussion

To increase muscle mass in Chinese domestic yellow cattle, we introduced specific *MSTN* mutations using ZFN-mediated mutagenesis combined with SCNT as an alternative to conventional crossing breeding. We tested three pairs of *MSTN*-specific ZFNs in bovine fibroblasts, and their mutation efficiencies ranged from 0.85% (set 2) to 14.52% (set 1) (Table S1), providing indirect evidence that ZFNs may not be highly active in the complex milieu of the eukaryotic nucleus.

The ZFNs targeted the same loci in different bovine cell lines, and exhibited similar gene disruption efficiencies, suggesting a high level of ZFN target sequence specificity (Table S2). Exhibiting a targeting efficiency up to 20% (Table 1), our data indicate that our ZFN-based method is an efficient, convenient, and time-saving technique for gene knockout. We introduced biallelic mutations in cells using a one-step approach that effectively shortened the breeding cycle of the genetically modified animal.

In most of the ZFN-induced *MSTN* mutant cells, we detected the expression of processed N-terminal MSTN protein. However, MSTN was not expressed in the cells of colony 6, suggesting that colony 6 represented the most optimal donor cell candidate for SCNT (Figure 3B). In the other mutant colonies, although the *MSTN* ORF was disrupted in one or both alleles, the altered protein was expressed. The ZFN cleavage site was located in the upstream region of the splicing signal sequence in exon 1 of *MSTN* (Table S1).

In the cells of colony 6, the splicing signal sequence was disrupted in both alleles, which may have abrogated the expression of *MSTN* mRNA. Both alleles were also disrupted in the cells of colony 7. The splicing signal remained intact in the cells of colonies 7, 20, and 44, which may have permitted the production of the protein. MSTN expression was upregulated in colonies 7 and 20 (Figure 3B), with colony 7 exhibiting the highest level of expression (Figure 3A). The exact reason for this is unclear, but we speculate that the truncated, non-functional protein product might induce negative feedback regulation of MSTN expression. Our analysis of the cellular levels of the MSTN mRNA and protein provide important information for studies of *MSTN* gene function and somatic cell cloning.

Our study is the first to generate *MSTN* mutations in cattle using cutting-edge biotechnology based on ZFNs, and both of the cloned *MSTN* mutant calves exhibited the double-muscled phenotype at one month of age (Figure 4B). Previous research has shown that the 11-bp deletion in exon 3 of the *MSTN* gene in Belgian blue cattle causes a frameshift after codon 274, which results in a premature stop codon at codon 288 [5], [6], [9]. This 11-bp deletion allele expresses only one type of MSTN transcript, which also contains the 11-bp deletion. In the *MSTN* gene of Piedmontese cattle, a G-to-A point mutation in exon 3 introduces a tyrosine residue in place of a highly conserved cysteine residue (the fifth in a series of nine cysteines) in the MSTN protein [5], [24]. The *MSTN* mutations in the Belgian blue and Piedmontese cattle breeds, both of which display the double-muscled phenotype, are located in the C-terminal domain of the MSTN protein.

In our *MSTN* mutant calves, we found that the 6-bp deletion comprising one allele produced three variants of abnormally spliced mRNAs that resulted in the production of truncated forms of the MSTN protein which lacked the original C-terminal domain (Figure 5A). By contrast, the 117-bp deletion and 9-bp insertion that comprised the other allele resulted in a single amino acid substitution (Figure 5A). These results are consistent with those of the ELISA analysis, which showed that the level of MSTN protein with an intact C-terminal domain decreased by approximately 50% (Figure 6).

The double-muscled phenotype was observed in the mutant calves, and we will continue to monitor them to evaluate the effects of these *MSTN* mutations on muscle mass. In addition, the birth rate for calves with the ZFNs-induced *MSTN* mutations was higher than that of the other groups. This effect may have been caused by the use of mRNA in the transfection experiments, rather than plasmid DNA, and the drug-free cell culture medium (Table S5). However, Mendias et al. (2008) reported that the tendons of myostatin-deficient mice were small, brittle, and hypocellular. Thus, we will perform a long-term evaluation of the tendons of the *MSTN* mutant cattle and their offspring to assess the long-term effects of the *MSTN* mutations.

We used ZFN-induced gene modification in bovine fibroblast cells to introduce mutations in *MSTN*, with a high level of specificity. ZFN-based mutagenesis is a promising new methodology for studying gene function, and may be applied to the biotech industry. In our current study, the high efficiency of ZFN-induced gene knockout provided a rapid and biologically safe single-step method of marker-gene-free site-specific mutagenesis. It will open a new era of genetically modified farm animals. We are currently planning to extend the application of ZFN-induced *MSTN* gene knockout to Aberdeen Angus cattle.

## Materials and Methods

### Ethics Statement

Our study protocols were approved by the Committee on the Ethics of Animal Experiments at China Agricultural University (Beijing, China), and all the procedures were performed in strict accordance with the *Guide for the Care and Use of Laboratory Animals*. The owners of the Chinese domestic yellow cattle consented to the use of their animals in our study. All surgery was performed under sodium pentobarbital-induced general anesthesia, and all efforts were made to minimize animal suffering.

### Generation of ZFN mRNA

The plasmids encoding the MSTN-ZFNs were linearized by digestion with *Xba*I (Takara-Bio, Shiga, Japan). The ZFN mRNA was transcribed in vitro from 1 µg of DNA template using the Ambion mMESSAGEmMACHINE T7 mRNA Transcription kit (Life Technologies, Carlsbad, CA, USA) and the Ambion Poly(A) Tailing kit (Life Technologies), and the MSTN-ZFN mRNA was purified using the Ambion MEGAClear kit (Life Technologies), according to the manufacturer’s instructions. The mRNA was quantified using a Nanodrop spectrophotometer (Thermo Fisher, Waltham, MA, USA). The purified RNA was diluted to 500 ng/µl for transfection, and stored at −80°C.

### Preparation, Culture, and Transfection of Bovine Primary Fibroblasts

The primary bovine fetal fibroblast (BFF) cell line was isolated from a Chinese domestic yellow cattle fetus (LXH, on day 38–40) by disaggregation of the whole body without the head and viscera. The cells were cultured in Gibco DMEM (Invitrogen, Carlsbad, CA, USA) supplemented with 10% fetal bovine serum (Invitrogen) at 37.5°C in 5% CO_2_ and humidified air. Passage-3 BFF cells were transfected with the MSTN-ZFN mRNAs using the Amaxa Nucleofector reagent (Lonza, Basel, Switzerland), according to program T-016 in the manufacturer’s guidelines. A ZFN monomer cotransfection ratio of 1∶1 was used to generate a high mutation frequency, and 4 µg of total mRNA was used to transfect 10^6^ cells. The cell colonies that formed 24 to 48 h after transfection were dispersed by limiting dilution to a cell concentration of 500 cells/dish (10 cm^2^). The cell clones were examined 6 to 7 days after limiting dilution using an inverted microscope and marked by drawing a circle around them on the bottom of the dish using a marking pen. Then, single-cell clones were isolated by trypsinization after placing a cloning cylinder around the colony, and sealing it with sterile silicone high vacuum grease. The cells of each clone were subsequently expanded, analyzed, and cryopreserved 12 to 14 days later.

### Surveyor Nuclease Assay to Detect ZFN-induced Mutations

Genomic DNA was extracted from a mixture of transfected cells at 24 h post-transfection. Cultured cells were homogenized in 600 µl of lysis buffer containing 10 mM Tris-HCl, 100 mM EDTA, 0.5% SDS, and 400 µg/ml proteinase K (pH 8.0), and the lysis mixture was incubated at 55°C for 2 h with vigorous shaking. Then DNA was isolated using phenol/chloroform extraction and ethanol precipitation. To detect the ZFN-induced *MSTN* mutations, we amplified DNA sequences of the genomic DNA from mixed cells using PCR. The 202 U forward primer (5′-GAATGAGAACAGCGAGCAG-3′) and 948 L reverse primer (5′-ATAGGCTTCAACCTCTACAGA-3′) were used to amplify a 767-bp sequence flanking the ZFN target site. Thermal cycling was performed at 94°C for 10 min, followed by 35 cycles of 94°C for 1 min, 60°C for 30 sec, and 72°C for 40 sec, with a final extension at 72°C for 10 min. The PCR products were digested using the Surveyor nuclease provided in the Surveyor Mutation Detection kit (Transgenomic, Omaha, NE, USA), according to the manufacturer’s protocol. The DNA bands were quantified using the ImageJ software (National Institutes of Health, USA).

### Sequence Analysis of ZFN Target Site in *MSTN*

The genomic DNA of single-cell colonies was isolated by phenol/chloroform extraction and ethanol precipitation. To characterize the mutation at the targeted site, we PCR amplified *MSTN* gene sequence from both mixed cells and single-cell colonies using the 202 U/948 L primer pair, as described above. The PCR product derived from the mixed cells was TA-cloned and sequenced, and the gene disruption efficiency was calculated. The PCR products derived from the single-cell colonies were sequenced directly. If double peaks were observed in the chromatogram to the right of *Fok*I recognition sequence, the respective PCR product was TA-cloned and sequenced. The sequences were aligned using DNAman (Lynnon, Quebec, Canada).

### RT-PCR Analysis of MSTN mRNA

Total RNA was extracted using the Trizol reagent (Invitrogen) and chloroform, and the RNA was quantified using a Nanodrop spectrophotometer. First-strand cDNAs of *MSTN* and *GAPDH* (endogenous control) were generated by RT using 1 µg of total RNA and oligo-dT primers. The *MSTN* cDNA was PCR amplified using the M-F (5′-TACAAGGTATACTGGAATCCGATCTCT-3′) and M-R (5′-TGACCATTCTCATCTAAAGCTTTGA-3′) oligonucleotide primers. The *GAPDH* cDNA was PCR amplified using the G-F (5′-GCAAGTTCCACGGCACAG-3′) and G-R (5′-CGCCAGTAGAAGCAGGGAT-3′) primers. Thermal cycling was performed using 35 cycles of denaturation at 94°C for 30 sec, annealing at 60°C for 30 sec, and elongation at 72°C. The size of the RT-PCR products were estimated by electrophoresis of a 5-µl aliquot on a 2.0% agarose gel.

### Western Blotting of N-terminal MSTN Protein

Total protein was extracted using the IP lysis buffer (Beyotime, Beijing, China), and the protein was quantified using an Infinite 200 PRO multimode reader (Tecan, Männedorf, Switzerland). An aliquot containing 50 µg of total protein was subjected to SDS-PAGE on a 12% acrylamide gel, and the proteins bands were electrophoretically transferred to an Amersham Hybond TM-N^+^ membrane (GE Healthcare, Waukesha, WI, USA). The N-terminal domain of MSTN, β-actin, and tubulin proteins were detected using a 1∶2000 dilution of an anti-MSTN (LifeSpan Biosciences, Seattle, WA, USA), anti-β-actin (Santa Cruz Biotechnology, Dallas, TX, USA), or anti-tubulin (Abcam, Cambridge, MA, USA) primary antibody, and primary antibody reactivity was detected using a 1∶5000 dilution of a horseradish peroxidase-conjugated secondary antibody (Santa Cruz Biotechnology). The N-terminal domain of MSTN, β-actin, and tubulin protein bands were visualized using an enhanced chemiluminescence method (Thermo Scientific).

### Somatic Cell Nuclear Transfer

The SCNT was performed, as described previously [25], [26]. The *MSTN* mutant cell colonies (donors) were transferred into enucleated oocytes to produce reconstructed embryos that were then electrically fused using a BTX 2001 Electro Cell Manipulator (BTX, San Diego, CA, USA). The reconstructed embryos were activated by treatment with cycloheximide (10 mg/ml) and cytochalasin-D (2.5 mg/ml) in CR1aa culture medium [27] containing 0.1% (w/v) bovine serum albumin for 1 h, followed by incubation in CR1aa medium containing 10 mg/ml cycloheximide for 4 h. On day 7, two or three high quality reconstructed blastocysts were transferred to each synchronous recipient cow, and the gestation of the recipients was examined on days 60, 90, and 240 post-transfer.

### *MSTN* mRNA Sequencing

Total RNA was extracted from samples of the quadriceps of *MSTN* mutant and WT calves using the Trizol reagent and chloroform, and the RNA was quantified using a Nanodrop spectrophotometer. RT was performed using 1 µg of total RNA and oligo-dT primers to generate the cDNA. The cDNA encoding the MSTN mRNA sequence was PCR amplified using the 5′ UTR (5′-TTTGGCTTGGCGTTACTCAAAAG-3′) and 3′ UTR (5′-TACTCTAGGCTTATAGCCTGTGGT-3′) primers. Thermal cycling was performed using 35 cycles of denaturation at 94°C for 30 sec, annealing at 60°C for 30 sec, and elongation at 72°C. The PCR products were TA-cloned and sequenced to characterize the ZFN-induced mutations. The MSTN mRNA sequences were aligned using the DNAman software.

### Analysis of C-terminal MSTN Protein

Total protein was extracted from samples of the quadriceps of *MSTN* mutant and WT calves using the IP lysis buffer, and the protein was quantified using an Infinite 200 PRO multimode reader. The amount of MSTN protein with a C-terminal domain was determined using an ELISA kit (Life Sciences Advanced Technologies, St. Petersburg, FL, USA), according to the manufacturer’s protocol.

### Statistical Analysis

Differences between the various data sets were evaluated using a chi-squared analysis and two-tailed Student’s *t*-tests. The results of comparisons with *P*<0.05 were considered to represent statistically significant differences. The statistical analysis was performed using the SAS software (SAS Institute, Cary, NC, USA).

## Supporting Information

Figure S1 **Surveyor nuclease assay.** Restriction enzyme digestion of the *MSTN* PCR product derived from mixed bovine fibroblasts after MSTN-ZFN-mRNA transfection for 24 h. Multiple bands indicate a mutation (deletions or/and insertions) occurred in *MSTN*. The intensity analysis of the bands indicated that the ZFNs cut the target DNA sequence with high efficiency (≥5%). M: 100-bp marker ladder; NC: negative-control group; ZT: ZFN-transfected group.
(DOC)

Figure S2 **RT-PCR analysis of the mRNA levels of** ***MSTN*** **and various** ***MSTN*** **signaling pathway related factors.** The expression of the *P21* and *Myf5* mRNAs were downregulated, whereas the expression of *Myogenin* was upregulated. The level of *MSTN* mRNA in the mutant cells was a lower than that of the WT cells. *GAPDH* was used as a loading control.
(DOC)

Figure S3 **Western blotting analysis of processed mature N-terminal MSTN protein.** The expression of the processed mature N-terminus of the MSTN protein was upregulated in the *MSTN* mutant calves, compared to that of the WT calves, which may have been due to negative feedback regulation induced by the truncated protein. “+55 bp” indicates the monoallelic mutation, which consisted of a 55-bp insertion in *MSTN*. Total protein (50 µg) from quadraceps muscle of each calf was subjected to SDS-PAGE on a 12% acrylamide gel, and the processed mature N-terminus of the MSTN protein was detected using a mouse anti-myostatin antibody. Tubulin was used as a loading control. The (+) symbol represents an insertion, and the (−) symbol represents a deletion.
(DOC)

Table S1 **Comparing the efficiency of different ZFN constructs for** ***MSTN*** **loci in bovine fibroblast cells.** *The mutation efficiency was calculated as the mutant TA-cloning of PCR products of mixed cells divided by the total sequencing number.
(DOC)

Table S2 **Comparison of the mutation efficiency of the set-1 ZFNs in different bovine fibroblast cell lines.** *The mutant efficiency was calculated as the mutant TA-cloning of PCR products of mixed cells divided by the total sequencing number.
(DOC)

Table S3 **Characterization of the ZFN-induced mutations in** ***MSTN.*** The ZFNs were spaced 5 bp apart (lowercase letters), and most of the mutations included a 5-bp insertion/deletion at the target site. This indicates the use of ZFNs spaced 5 bp apart typically results in 5-bp 5′ overhangs at the cleaved ends of the double-stranded DNA.
(DOC)

Table S4 **Genotype analysis of ZFN-induced** ***MSTN*** **mutations in bovine fibroblast colonies.** Underlined bases indicate ZFN binding sites and the spacer nucleotides (red letters) were the cleavage sites. Colonies 6 and 7 had the biallelic *MSTN* mutations. Colonies 20 and 44 had a monoallelic *MSTN* mutation.
(DOC)

Table S5 **Somatic cell nuclear transfer and embryo transplant data.** LXH-MSTN was the ZFN-induced *MSTN* gene knockout. 094-BLG was the ZFN-induced *BLG* gene modification. LXH-FST was a follistatin transgene. Xiangwa was a human lactoferrin transgene. 094-NEO was a neomycin transgene. 094-CD20 was a CD20 transgene. The data from our lab used as the control.
(DOC)

Table S6 **Primers used to examine the off-targeting effect of the** ***MSTN*** **-specific ZFNs.**
(DOC)

Table S7 **Analysis of the potential off-targeting effects of the ZFNs.** Similar target sequences were predicted using BLASTn, and 15 primer pairs were designed to PCR amplify and sequence regions containing 15 of the most similar target sequences. Except for the set-1 ZFN target sequence (the first line), none of the similar target sequences were mutated, suggesting that the ZFNs displayed a high level of specificity.
(DOC)

Checklist S1 **The ARRIVE (Animal Research: Reporting of In Vivo Experiments) Guidelines Checklist.**
(DOC)
