# Supplementary material for: Efficient Generation of Myostatin (MSTN) Biallelic Mutations in Cattle Using Zinc Finger Nucleases
Source: PLoS One. 2014 Apr 17;9(4):e95225. doi: 10.1371/journal.pone.0095225 (PMC3990601; doi:10.1371/journal.pone.0095225)
Supplement: Figure S1 — Surveyor nuclease assay. Restriction enzyme digestion of the MSTN PCR product derived from mixed bovine fibroblasts after MSTN-ZFN-mRNA transfection for 24 h. Multiple bands indicate a mutation (deletions or/and insertions) occurred in MSTN. The intensity analysis of the bands indicated that the ZFNs cut the target DNA sequence with high efficiency (≥5%). M: 100-bp marker ladder; NC: negative-control group; ZT: ZFN-transfected group. (DOC) [file pone.0095225.s001.doc]

**Figure S1**


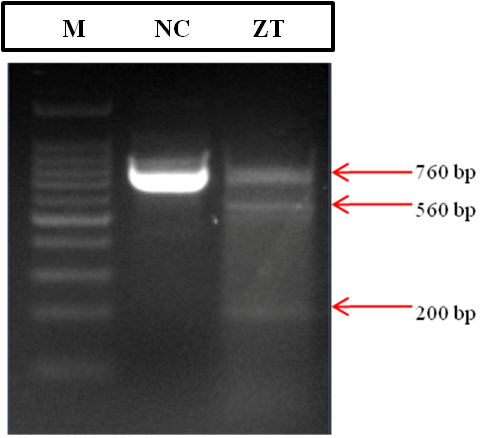


**Figure S1.** Surveyor nuclease assay. Restriction enzyme digestion of the *MSTN* PCR product derived from mixed bovine fibroblasts after MSTN-ZFN-mRNA transfection for 24 h. Multiple bands indicate a mutation (deletions or/and insertions) occurred in *MSTN*. The intensity analysis of the bands indicated that the ZFNs cut the target DNA sequence with high efficiency (≥5%). M: 100-bp marker ladder; NC: negative-control group; ZT: ZFN-transfected group.
