# Supplementary material for: Efficient Generation of Myostatin (MSTN) Biallelic Mutations in Cattle Using Zinc Finger Nucleases
Source: PLoS One. 2014 Apr 17;9(4):e95225. doi: 10.1371/journal.pone.0095225 (PMC3990601; doi:10.1371/journal.pone.0095225)
Supplement: Figure S2 — RT-PCR analysis of the mRNA levels of MSTN and various MSTN signaling pathway related factors. The expression of the P21 and Myf5 mRNAs were downregulated, whereas the expression of Myogenin was upregulated. The level of MSTN mRNA in the mutant cells was a lower than that of the WT cells. GAPDH was used as a loading control. (DOC) [file pone.0095225.s002.doc]

**Figure S2**


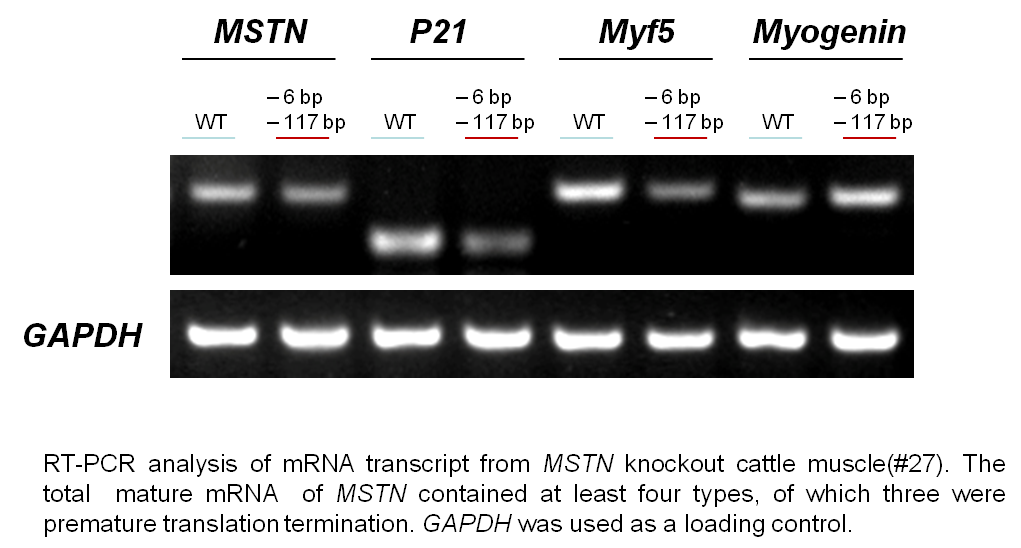


**Figure S2.** RT-PCR analysis of the mRNA levels of *MSTN* and various *MSTN* signaling pathway related factors. The expression of the *P21* and *Myf5* mRNAs were downregulated, whereas the expression of *Myogenin* was upregulated. The level of *MSTN* mRNA in the mutant cells was a lower than that of the WT cells. *GAPDH* was used as a loading control.
