# Supplementary material for: Efficient Generation of Myostatin (MSTN) Biallelic Mutations in Cattle Using Zinc Finger Nucleases
Source: PLoS One. 2014 Apr 17;9(4):e95225. doi: 10.1371/journal.pone.0095225 (PMC3990601; doi:10.1371/journal.pone.0095225)
Supplement: Figure S3 — Western blotting analysis of processed mature N-terminal MSTN protein. The expression of the processed mature N-terminus of the MSTN protein was upregulated in the MSTN mutant calves, compared to that of the WT calves, which may have been due to negative feedback regulation induced by the truncated protein. “+55 bp” indicates the monoallelic mutation, which consisted of a 55-bp insertion in MSTN. Total protein (50 µg) from quadraceps muscle of each calf was subjected to SDS-PAGE on a 12% acrylamide gel, and the processed mature N-terminus of the MSTN protein was detected using a mouse anti-myostatin antibody. Tubulin was used as a loading control. The (+) symbol represents an insertion, and the (−) symbol represents a deletion. (DOC) [file pone.0095225.s003.doc]

**Figure S3**


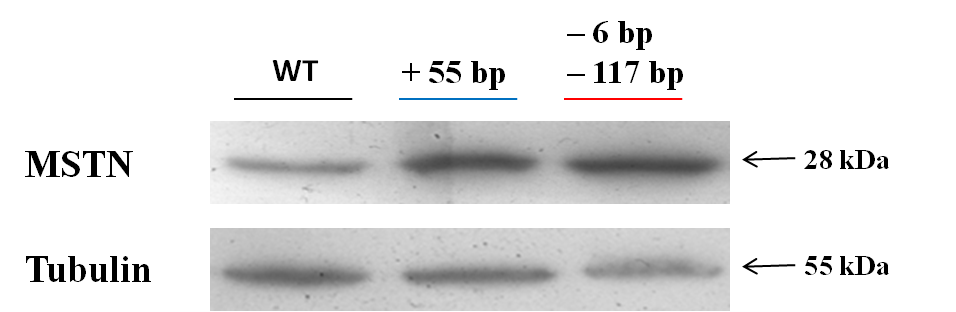


**Figure S3.** Western blotting analysis of processed mature N-terminal MSTN protein. The expression of the processed mature N-terminus of the MSTN protein was upregulated in the *MSTN* mutant calves, compared to that of the WT calves, which may have been due to negative feedback regulation induced by the truncated protein. “+ 55 bp” indicates the monoallelic mutation, which consisted of a 55-bp insertion in *MSTN*. Total protein (50 μg) from quadraceps muscle of each calf was subjected to SDS-PAGE on a 12% acrylamide gel, and the processed mature N-terminus of the MSTN protein was detected using a mouse anti-myostatin antibody. Tubulin was used as a loading control. The (+) symbol represents an insertion, and the (-) symbol represents a deletion.
