# Supplementary material for: Efficient Generation of Myostatin (MSTN) Biallelic Mutations in Cattle Using Zinc Finger Nucleases
Source: PLoS One. 2014 Apr 17;9(4):e95225. doi: 10.1371/journal.pone.0095225 (PMC3990601; doi:10.1371/journal.pone.0095225)
Supplement: Table S1 — Comparing the efficiency of different ZFN constructs for MSTN loci in bovine fibroblast cells. *The mutation efficiency was calculated as the mutant TA-cloning of PCR products of mixed cells divided by the total sequencing number. (DOC) [file pone.0095225.s004.doc]

**Table S1**

**Table S1.** Comparing the efficiency of different ZFN constructs for *MSTN* loci in bovine fibroblast cells.

| ZFN plasmid | Loci | Binding and targeting sequence | Mutation efficiency* |
| --- | --- | --- | --- |
| ZFN set 1 | Exon 1 & Intron1 | GTCATTACCATGCCCACGGagtgtGAGTAGTCCTGCTGGT | 17/117 (14.53%) |
| ZFN set 2 | Exon 2 | CTCATCAAACCCATGAaagacggTACAAGGTATACTGG | 1/117 (0.85%) |
| ZFN set 3 | Exon 2 | TTCCCAGAACcaggaGAAGATGGACTGGTA | 11/122 (9.02%) |

*The mutation efficiency was calculated as the mutant TA-cloning of PCR products of mixed cells divided by the total sequencing number.
