# Supplementary material for: Efficient Generation of Myostatin (MSTN) Biallelic Mutations in Cattle Using Zinc Finger Nucleases
Source: PLoS One. 2014 Apr 17;9(4):e95225. doi: 10.1371/journal.pone.0095225 (PMC3990601; doi:10.1371/journal.pone.0095225)
Supplement: Table S2 — Comparison of the mutation efficiency of the set-1 ZFNs in different bovine fibroblast cell lines. *The mutant efficiency was calculated as the mutant TA-cloning of PCR products of mixed cells divided by the total sequencing number. (DOC) [file pone.0095225.s005.doc]

**Table S2**

**Table S2.** Comparison of the mutation efficiency of the set-1 ZFNs in different bovine fibroblast cell lines.

| Cell line | Mutation | Total | Mutation efficiency* |
| --- | --- | --- | --- |
| LXH-LS | 17 | 117 | 14.53% |
| LXH-101 | 16 | 106 | 15.09% |
| LXH-102 | 15 | 107 | 14.02% |

*The mutant efficiency was calculated as the mutant TA-cloning of PCR products of mixed cells divided by the total sequencing number.
