# Supplementary material for: Efficient Generation of Myostatin (MSTN) Biallelic Mutations in Cattle Using Zinc Finger Nucleases
Source: PLoS One. 2014 Apr 17;9(4):e95225. doi: 10.1371/journal.pone.0095225 (PMC3990601; doi:10.1371/journal.pone.0095225)
Supplement: Table S3 — Characterization of the ZFN-induced mutations in MSTN. The ZFNs were spaced 5 bp apart (lowercase letters), and most of the mutations included a 5-bp insertion/deletion at the target site. This indicates the use of ZFNs spaced 5 bp apart typically results in 5-bp 5′ overhangs at the cleaved ends of the double-stranded DNA. (DOC) [file pone.0095225.s006.doc]

**Table S3**

**Table S3.** Characterization of the ZFN-induced mutations in *MSTN.*

| ZFN construct | Target sequence | 5 bp mutants / total mutation |
| --- | --- | --- |
| ZFN set 1 | GTCATTACCATGCCCACGGagtgtGAGTAGTCCTGCTGGT | 10/78 (12.8%) |
| ZFN set 3 | TTCCCAGAACcaggaGAAGATGGACTGGTA | 4/11 (36.4%) |

The ZFNs were spaced 5 bp apart (lowercase letters), and most of the mutations included a 5-bp insertion/deletion at the target site. This indicates the use of ZFNs spaced 5 bp apart typically results in 5-bp 5’ overhangs at the cleaved ends of the double-stranded DNA.
