# Supplementary material for: Efficient Generation of Myostatin (MSTN) Biallelic Mutations in Cattle Using Zinc Finger Nucleases
Source: PLoS One. 2014 Apr 17;9(4):e95225. doi: 10.1371/journal.pone.0095225 (PMC3990601; doi:10.1371/journal.pone.0095225)
Supplement: Table S4 — Genotype analysis of ZFN-induced MSTN mutations in bovine fibroblast colonies. Underlined bases indicate ZFN binding sites and the spacer nucleotides (red letters) were the cleavage sites. Colonies 6 and 7 had the biallelic MSTN mutations. Colonies 20 and 44 had a monoallelic MSTN mutation. (DOC) [file pone.0095225.s007.doc]

**Table S4**

**Table S4.** Genotype analysis of ZFN-induced *MSTN* mutations in bovine fibroblast colonies.


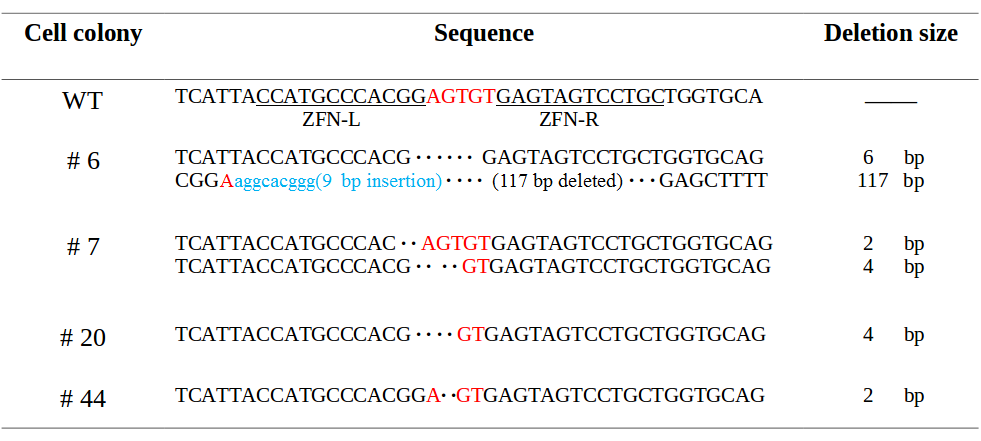


Underlined bases indicate ZFN binding sites and the spacer nucleotides (red letters) were the cleavage sites. Colonies 6 and 7 had the biallelic *MSTN* mutations. Colonies 20 and 44 had a monoallelic *MSTN* mutation.
