# Supplementary material for: Efficient Generation of Myostatin (MSTN) Biallelic Mutations in Cattle Using Zinc Finger Nucleases
Source: PLoS One. 2014 Apr 17;9(4):e95225. doi: 10.1371/journal.pone.0095225 (PMC3990601; doi:10.1371/journal.pone.0095225)
Supplement: Table S5 — Somatic cell nuclear transfer and embryo transplant data. LXH-MSTN was the ZFN-induced MSTN gene knockout. 094-BLG was the ZFN-induced BLG gene modification. LXH-FST was a follistatin transgene. Xiangwa was a human lactoferrin transgene. 094-NEO was a neomycin transgene. 094-CD20 was a CD20 transgene. The data from our lab used as the control. (DOC) [file pone.0095225.s008.doc]

**Table S5**

**Table S5.** Somatic cell nuclear transfer and embryo transplant data.

| Cell colony | Blastocysts | | Blastocyst % | Surrogates | Pregnant | Birth(%) | Survival(%) |
| --- | --- | --- | --- | --- | --- | --- | --- |
| LXH-MSTN | 1336 | 49.5±2.3 | | 123 | 35 | 18 (51.4) | 3 (16.7) |
| 094-BLG | 643 | 57.1±1.4 | | 99 | 41 | 8 (19.5) | 1 (12.5) |
| LXH-FST | 839 | 41.5±6.2 | | 153 | 60 | 20 (33.3) | 15 (75.0) |
| Xiangwa | 497 | 43.4±8.9 | | 158 | 19 | 3 (15.8) | 1(33.3) |
| 094-NEO | 462 | 42.3±8.5 | | 129 | 28 | 11 (39.3) | 5 (45.5) |
| 094-CD20 | 478 | 45.4±7.1 | | 61 | 11 | 4 (36.4) | 2 (50.0) |

LXH-MSTN was the ZFN-induced *MSTN* gene knockout. 094-BLG was the ZFN-induced *BLG* gene modification. LXH-FST was a follistatin transgene. Xiangwa was a human lactoferrin transgene. 094-NEO was a neomycin transgene. 094-CD20 was a CD20 transgene. The data from our lab used as the control.
