# Supplementary material for: Efficient Generation of Myostatin (MSTN) Biallelic Mutations in Cattle Using Zinc Finger Nucleases
Source: PLoS One. 2014 Apr 17;9(4):e95225. doi: 10.1371/journal.pone.0095225 (PMC3990601; doi:10.1371/journal.pone.0095225)
Supplement: Table S6 — Primers used to examine the off-targeting effect of the MSTN -specific ZFNs. (DOC) [file pone.0095225.s009.doc]

**Table S6**

**Table S6.** Primers used to examine the off-targeting effect of the *MSTN*-specific ZFNs.

| **Similar sequence location** | **Primer-F** | **Primer-R** |
| --- | --- | --- |
| chr18: 20233719-20233684 | TGTTCCCACAAATCCAGTCA | AGGCTGATTTCACAGGCACT |
| chr18: 19431643-19431674 | CATCATGGAAGCAGAAGCAA | TGCTGTATGAACCCAATCCA |
| chr9: 89095135-89095171 | GAAAGGTGGGTCCTGTCTGA | GGAAGAAAGGAGGGAAGGTG |
| chr13: 33255092-33255059 | TCCTCTTGGTTCCCTGACTG | GGTTTCTGCTGCTCTGAACC |
| chr22: 34484856-34484824 | GGCCCACCCTTTAGACAAAT | GGACAGAAATCCAGGACAGC |
| chr27: 2027449-2027480 | CCGTGACACAGAGGACAGAA | TAGAGTTTTGCGGGATTGCT |
| chr11: 9306858-9306888 | GGAGCTCGAATCCAAGAATG | GAGGCAGGTGAAGAGTGAGG |
| chr26: 36179237-36179269 | AATCTGGGAGTTGGTGATGG | GAAGTCACAAATGGGGGATG |
| chr14: 79307319-79307348 | TCAGGGACACCATTCAGACA | TCCCAACTCAGGGACTGAAC |
| chr24: 50109077-50109105 | AGGGAGCAGAATTAGCAGGA | AATGAGATGCAGACGCTGTG |
| chr4: 40411441-40411472 | GACCAGTTGGAGGTGATGCT | ACCCTGTTCCTCAGTGTTGG |
| chr10: 82662969-82662996 | GAGCCAAGTCCCACACAAAT | CAACCCCAAACGAGAAACAT |
| chr17: 26986176-26986146 | TTTACAGTCCAAATGTGAGCTAGG | TCTCATTGGCTGAGCTGTTG |
| chr2: 112596352-112596323 | AAGCTTTTGGGGCAGATACA | GGTGTTTGGTGAAAGGGAAA |
| chr8: 97276582-97276610 | ATGAAGGGAAGCACGTTGAC | GCTGAGAGGGATCTGAATGG |
