# Supplementary material for: Efficient Generation of Myostatin (MSTN) Biallelic Mutations in Cattle Using Zinc Finger Nucleases
Source: PLoS One. 2014 Apr 17;9(4):e95225. doi: 10.1371/journal.pone.0095225 (PMC3990601; doi:10.1371/journal.pone.0095225)
Supplement: Table S7 — Analysis of the potential off-targeting effects of the ZFNs. Similar target sequences were predicted using BLASTn, and 15 primer pairs were designed to PCR amplify and sequence regions containing 15 of the most similar target sequences. Except for the set-1 ZFN target sequence (the first line), none of the similar target sequences were mutated, suggesting that the ZFNs displayed a high level of specificity. (DOC) [file pone.0095225.s010.doc]

**Table S7**

**Table S7.** Analysis of the potential off-targeting effects of the ZFNs.

| **Sequence location** | **Alignment length** | **Mismatch** | **Gap opens** | **Identity (%)** | **Mutation** |
| --- | --- | --- | --- | --- | --- |
| chr2: 6214050-6214089 | 40 | 0 | 0 | 100.0 | Yes |
| chr18: 20233719-20233684 | 39 | 7 | 1 | 74.4 | No |
| chr18: 19431643-19431674 | 37 | 3 | 2 | 75.7 | No |
| chr9: 89095135-89095171 | 37 | 9 | 0 | 75.7 | No |
| chr13: 33255092-33255059 | 34 | 5 | 2 | 79.4 | No |
| chr22: 34484856-34484824 | 34 | 7 | 1 | 76.5 | No |
| chr27: 2027449-2027480 | 34 | 5 | 2 | 79.4 | No |
| chr11: 9306858-9306888 | 33 | 5 | 2 | 78.8 | No |
| chr26: 36179237-36179269 | 33 | 7 | 0 | 78.8 | No |
| chr14: 79307319-79307348 | 32 | 4 | 1 | 81.3 | No |
| chr24: 50109077-50109105 | 32 | 4 | 1 | 78.1 | No |
| chr4: 40411441-40411472 | 32 | 5 | 1 | 78.1 | No |
| chr10: 82662969-82662996 | 31 | 3 | 2 | 80.7 | No |
| chr17: 26986176-26986146 | 31 | 6 | 1 | 77.4 | No |
| chr2: 112596352-112596323 | 31 | 4 | 2 | 77.4 | No |
| chr8: 97276582-97276610 | 30 | 5 | 1 | 80.0 | No |

Similar target sequences were predicted using BLASTn, and 15 primer pairs were designed to PCR amplify and sequence regions containing 15 of the most similar target sequences. Except for the set-1 ZFN target sequence (the first line), none of the similar target sequences were mutated, suggesting that the ZFNs displayed a high level of specificity.
